# Combined HBsAg and anti-HBc testing is required to estimate hepatitis B virus seroprevalence in a low-endemic country: findings from a nationwide population-based serosurvey, Belgium, 2020

**DOI:** 10.2807/1560-7917.ES.2026.31.6.2500533

**Published:** 2026-02-12

**Authors:** Arno Furquim d’Almeida, Erwin Ho, Christian Schüttler, Philippe Beutels, Niel Hens, Sandra Dudareva, Pierre Van Damme, Heidi Theeten, Thomas Vanwolleghem

**Affiliations:** 1 Viral Hepatitis Research Group, Laboratory of Experimental Medicine and Pediatrics, University of Antwerp, Antwerp, Belgium; 2 Department of Gastroenterology and Hepatology, Antwerp University Hospital, Antwerp, Belgium; 3 Institute of Medical Virology, National Reference Centre for Hepatitis B Viruses and Hepatitis D Viruses, Justus Liebig University, Giessen, Germany; 4 Centre for Health Economics Research and Modelling of Infectious Diseases (CHERMID), Vaccine & Infectious Disease Institute (VAXINFECTIO), University of Antwerp, Antwerp, Belgium; 5 Data Science Institute, Interuniversity Institute for Biostatistics and statistical Bioinformatics (I-BioStat), University of Hasselt, Hasselt, Belgium; 6 Department of Infectious Disease Epidemiology, Robert Koch Institute, Berlin, Germany; 7 Institute of Public Health, Riga Stradins University, Riga, Latvia; 8 Centre for the Evaluation of Vaccination, Vaccine and Infectious Disease Institute (VAXINFECTIO), University of Antwerp, Antwerp, Belgium

**Keywords:** Hepatitis B virus, public health, screening, HBV prevalence, serosurvey

## Abstract

**BACKGROUND:**

The World Health Organization aims to eliminate hepatitis B virus (HBV) by 2030 through reducing incidence and mortality. Accurate prevalence estimates are crucial to guide policies and monitor progress towards HBV elimination. However, HBV prevalence can be overestimated when relying solely on hepatitis B surface antigen (HBsAg) because of unconfirmed or false-positive results. Robust screening algorithms to improve diagnostic accuracy and minimise false positives are required.

**AIM:**

We conducted a nationwide, population-based serosurvey to estimate HBV prevalence in Belgium by using HBsAg alone or combined with hepatitis B core antibody (anti-HBc) positivity as infection criterion.

**METHODS:**

We analysed HBsAg and anti-HBc in a total of 4,955 left-over serum samples from severe acute respiratory syndrome coronavirus 2 sero-epidemiology studies in 2020. Samples were stratified per region, 10-year age band and sex. A confirmatory anti-HBc neutralisation assay was performed in discordant samples.

**RESULTS:**

We detected HBsAg in 0.75% (37/4,955) of samples, of which 62.2% (23/37) were anti-HBc-negative and showed no specific anti-HBc signal in the neutralisation assay. None of the samples from ≤ 5-year-olds (n = 87) were double-positive. Weighted analysis estimated HBsAg seroprevalence at 0.74% (95% confidence interval (CI): 0.50–1.04). However, considering double HBsAg and anti-HBc positivity, an HBV prevalence of 0.25% (95% CI: 0.13–0.42) was retained. The HBsAg/anti-HBc prevalence in ≤ 33-year-olds was lower than in older adults (0.079% vs 0.36%; p = 0.015), consistent with Belgium’s vaccination policy.

**CONCLUSION:**

This serosurvey reinforces the importance of confirmatory anti-HBc testing in HBsAg-positive samples, particularly in low-endemic countries. Incorporating anti-HBc testing improves the correctness of prevalence estimates.

**Key public health message**

**What did you want to address in this study and why?**

We examined how many people in Belgium are infected with hepatitis B virus (HBV), as the last study dates from more than 20 years ago. We wanted to understand whether Belgium is on track to eliminate hepatitis B, as set forth by the World Health Organization. We looked at different ways of testing, because some tests might give a false-positive result. By using different infection criteria, we can estimate the number of infected people most accurately.

**What have we learnt from this study?**

We tested almost 5,000 samples from across Belgium, using two tests. We found that by using the combination of the two tests, a more accurate and reliable result is obtained than when relying on a single test only. We found that the number of people infected with HBV in Belgium is very low (0.25%), and that the childhood HBV vaccination implemented in Belgium in 1999 is effective to decrease the number of infections.

**What are the implications of your findings for public health?**

Using just one test can overestimate the number of HBV infections. With a combination of two tests, we get a more accurate and reliable result and both tests should be used in future studies. The results show that Belgium is a country where hepatitis B is rare. They help guide public health policies, track how well the HBV vaccination programme works, and give an idea how well Belgium is doing in controlling and eliminating the disease.

## Introduction

With ca 254 million people chronically infected and 1.2 million new infections annually, hepatitis B virus (HBV) remains an important global health concern [1]. Chronic HBV infection increases the risk of cirrhosis, hepatic decompensation, and hepatocellular carcinoma, which may eventually lead to liver-related mortality [2,3]. In 2022, the World Health Organization (WHO) estimated 1.1 million HBV-related deaths, marking an important increase compared with 2019. As such, viral hepatitis ranked, together with tuberculosis, as the second leading cause of death among communicable diseases that year [1].

There is a large regional variation of the burden of HBV infection, shaped by a complex interplay of cultural, socioeconomic and healthcare-related factors. High-endemic regions, such as the African and Western Pacific WHO Regions, bear a substantially higher burden due to high rates of mother-to-child transmission, a larger pool of chronic carriers, and limited and/or relatively late access to HBV vaccines in a context of under-resourced healthcare systems. In contrast, low-endemic regions, characterised by an HBV prevalence < 2% in the general population, such as Western Europe and North America, have achieved substantial reductions in HBV prevalence through widespread immunisation programmes and effective public health interventions [4,5].

However, global migration and population movements are reshaping HBV epidemiology in these low-endemic regions. It is reported that migrants and refugees, often originating from high-endemic regions, have higher HBV infection rates compared with the native population [6-9]. In 2016, it has been estimated that migrants from outside the European Union (EU) account for ca 25% of chronic hepatitis B cases in the EU [10]. These shifts highlight the value of targeted screening approaches and public health interventions. Ensuring equitable access to screening, vaccination, treatment and healthcare is essential not only for improving health outcomes in these vulnerable groups, who are often under-served by health systems and face social exclusion, stigma, discrimination and socioeconomic constraints, as well as language and health-literacy challenges [11], but also for reducing HBV transmission and the overall disease burden.

The WHO has set an ambitious target to eliminate viral hepatitis as a public health problem by 2030. This global initiative strives to reduce the HBV incidence by 95% and the HBV-related mortality by 65%, compared with the 2015 baseline. To achieve this, the WHO has proposed several absolute impact targets, focusing on systematic screening, prevention of mother-to-child transmission, universal vaccination and treatment optimisation [12]. For instance, specifically for the WHO European Region, the target is to achieve a hepatitis B surface antigen (HBsAg) seroprevalence of ≤ 0.1% in vaccinated cohorts, diagnosing and treating, respectively, at least 90% and 80% of people living with HBV, having at least 95% vaccination coverage of the third dose of childhood HBV vaccination, and screening > 95% of pregnant women for HBsAg [13,14].

A comprehensive understanding of the HBV prevalence, supported by accurate region-specific epidemiological data, is essential for designing effective public health policies, prioritising resources, monitoring the progress towards the WHO elimination goals, and informing other indicators of HBV elimination, such as the cascade of care. Population-based serosurveys offer valuable insight into the overall HBV burden in the population and provide an essential framework for monitoring elimination efforts.

Serosurveys typically rely on the detection of HBsAg as a marker for HBV infection [4,5]. While commercial automated assays are widely used in clinical practice, they are designed to be oversensitive, resulting in an increased risk for false-positive results [15]. Particularly in low-endemic countries, HBsAg false positivity remains a challenge in HBV prevalence estimation, as a few false positives may substantially inflate HBV prevalence estimates. False-positive HBsAg results have been reported to occur more frequently in younger individuals, females and in samples with low HBsAg levels [16]. It is, therefore, recommended to perform a confirmatory neutralisation assay in low-positive cases [17]. However, the widespread implementation of these confirmatory assays is hampered by limited availability and increased costs. Moreover, this approach would consume a considerable amount of serum which is not always feasible in studies using archived residual samples. This highlights the importance of robust screening algorithms determining when additional testing is required to improve diagnostic accuracy and minimise the risk for false-positive results.

In this study, we examine the impact of using HBsAg positivity alone compared with combined anti-HBc and HBsAg positivity for estimating HBV prevalence in a low-endemic country. In addition, we provide an updated national estimate of HBV prevalence for Belgium.

## Methods

### Study design

In this prospective cross-sectional nationwide serosurvey, we analysed left-over serum samples of collection periods 4 (8–13 June 2020), 5 (29 June–4 July 2020) and 6 (7–12 September 2020) of the study described by Herzog et al. [18]. Briefly, that study established a serum bank covering all Belgian regions by collecting residual sera from 10 high-throughput private routine clinical laboratories. The number of samples was allocated per age group (10-year age bands), per region (Brussels, Flanders and Wallonia) and per collection period, and was stratified by sex within each age group. All samples originated from ambulatory patients visiting their primary care physician for any reason. To avoid disproportionate selection, samples from hospitals and severe acute respiratory syndrome coronavirus 2 (SARS-CoV-2) triage centres were excluded. Therefore, the sample population is considered representative of the general Belgian population, as sampling did not involve hospitals, hepatology departments or infectious disease clinics, where individuals with HBV infection would be expected to be overrepresented. In the current study we analysed the samples from collection periods outside Belgian SARS-CoV-2 lockdown periods, thereby assuming to be periods with normal health-seeking behaviour. 

We performed the analytical tests in two phases. The initial phase included exclusively samples originating from adults, in line with the objective to estimate the HBV prevalence in the adult population. In a second phase, we assessed the HBV prevalence in children and adolescents (< 18 years-old). However, given the anticipated low prevalence of HBV in children and adolescents, analysis of all available samples was deemed necessary, resulting in an intentional oversampling of the 10–19-year-old age group. We assessed the HBV seroprevalence in children ≤ 5 years-old separately, as this is a key WHO impact target for HBV elimination. All samples were anonymised and had minimal demographical data available: sample date, age, sex and postal code of the place of residence.

### Analytical tests

We analysed HBsAg and hepatitis B core antibodies (anti-HBc) on automated analysers (Abbott Alinity I). A signal-to-cutoff (S/CO) ≥ 1.0 for anti-HBc and HBsAg was considered positive. We retested all HBsAg-positive results for the adult population (≥ 18 years-old) in duplicate. For the < 18-year-olds, there was insufficient volume to retest the HBsAg-positive samples in duplicate. For HBsAg-positive anti-HBc-negative samples, a non-commercial anti-HBc neutralisation assay was performed by the German National Reference Centre for Hepatitis B Viruses and Hepatitis D Viruses (Justus Liebig University, Giessen, Germany) to exclude false negativity as described by Huzly et al. [19].

### Statistical analyses

We used the Mann–Whitney U test to compare differences in HBsAg S/CO values. We weighted the samples to represent the Belgian population structure of 2020. We computed the weights by comparing sample and population frequencies by sex, age (10-year age bands) and province. We trimmed the weights to a maximum value of 3 to reduce the influence of samples in under-represented strata. One sample had missing information about the postal code and could therefore not be weighted. This sample was omitted from the weighted analyses. We used the Rao–Scott scaled chi-squared distribution, a survey-adjusted modification of the Pearson chi-squared test that accounts for differences between observed and expected frequencies and is preferred in survey analysis because it corrects for survey features such as weighting to provide valid statistical inferences [20]. All analyses were done with the statistical software R (version 4.4.1, R Foundation, Vienna, Austria) [21]. The R package ‘survey’ (version 4.4) was used for weighting and weighted analyses.

## Results

We analysed a total of 4,955 serum samples. A detailed overview of the regional, age and sex distribution is shown in Table 1. In Supplementary Figure S1 we additionally append the sample population compared with the 2020 Belgian population distribution.

**Table 1:** Regional, age and sex distribution of the sample population, hepatitis B virus serosurvey, Belgium, 2020 (n = 4,955)

Sample population	n	%
**Region** ^a^		
Brussels	443	8.9
Flanders	2,257	45.5
Wallonia	2,254	45.5
**Age group (years)**		
< 10	291	5.9
10–19	1,026	20.7
20–29	574	11.6
30–39	578	11.7
40–49	580	11.7
50–59	580	11.7
60–69	587	11.8
70–79	305	6.2
80–89	238	4.8
≥ 90	196	4.0
**Sex**		
Male	2,380	48.0
Female	2,575	52.0

^a^ One sample had missing information about the postal code.

### HBsAg and anti-HBc results in the sample population

**Figure 1:**
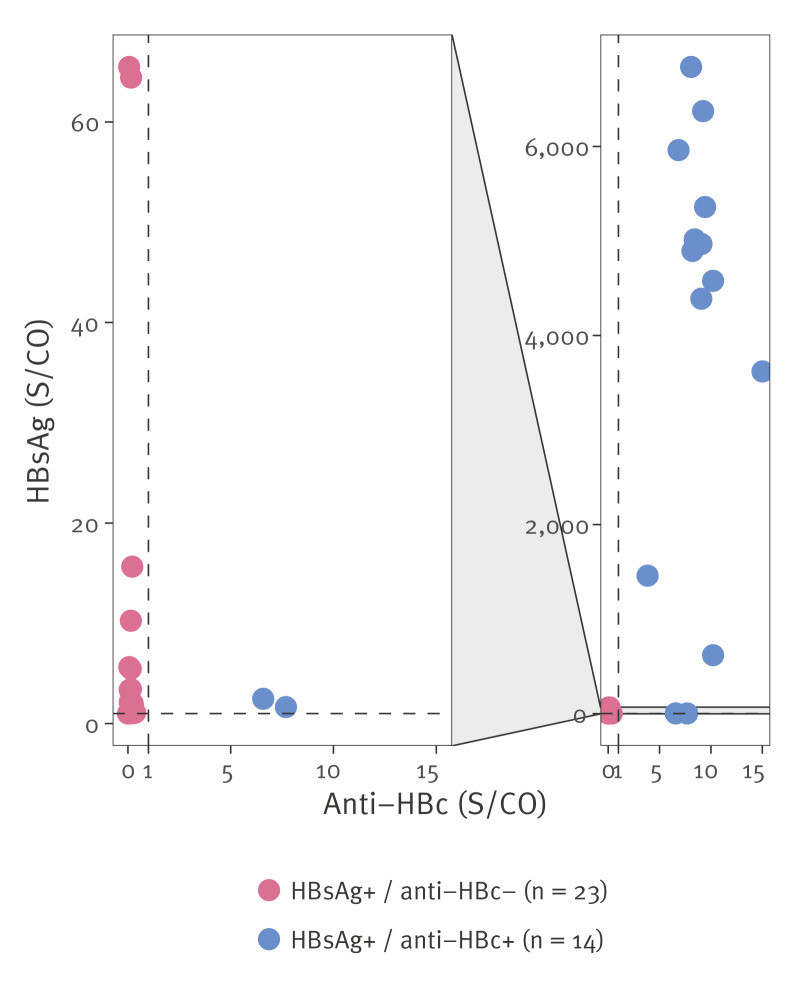
Signal-to-cutoff values of double-positive and HBsAg-positive anti-HBc-negative samples in the sample population, Belgium, 2020 (n = 4,955)

Anti-HBc: hepatitis B core antibodies; HBsAg: hepatitis B surface antigen; S/CO: signal-to-cutoff.

We detected HBsAg in 37 of the 4,955 samples (0.75%). Of these 37, 14 were anti-HBc-positive and 23 were anti-HBc-negative (Table 2). The HBsAg-positive anti-HBc-negative samples had significantly lower HBsAg S/CO ratios in comparison with the double-positive samples (median = 2.00 (IQR = 3.26) S/CO vs median = 4,737 (IQR = 3,274) S/CO; p < 0.001) (Figure 1). To confirm true anti-HBc negativity in these 23 samples, we performed an additional independent non-commercial anti-HBc neutralisation assay. In this assay, no specific anti-HBc signal was detected in any of the samples. Furthermore, 169 of the 4,955 (3.41%) samples were HBsAg-negative anti-HBc-positive with the commercial assays (Table 2).

**Table 2:** Hepatitis B virus markers in the sample population, Belgium, 2020 (n = 4,955)

HBsAg	Anti-HBc	Sample population	
		n	%
+	+	14	0.28
+	-	23	0.46
-	+	169	3.41
-	-	4,749	95.84

Anti-HBc, hepatitis B core antibodies; HBsAg, hepatitis B surface antigen.

### Age and sex distribution of HBsAg and anti-HBc-positive samples

**Figure 2:**
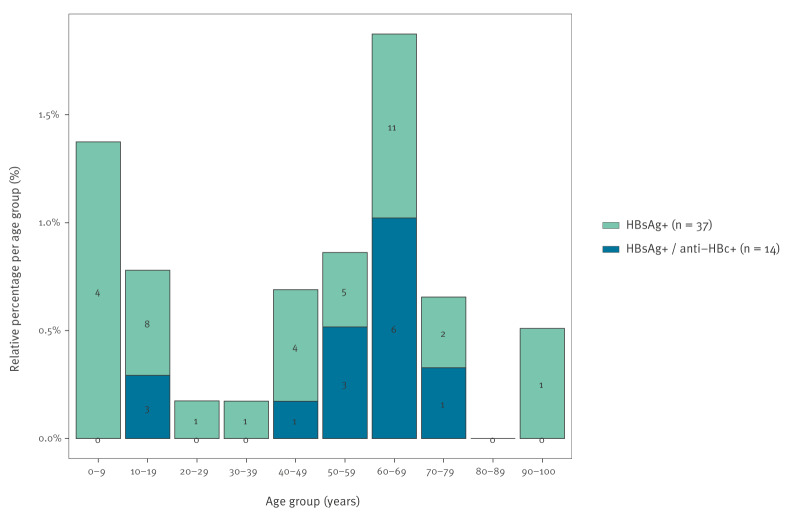
HBsAg-positive and double HBsAg anti-HBc-positive samples across age groups, Belgium, 2020 (n = 4,955)

Anti-HBc: hepatitis B core antibodies; HBsAg: hepatitis B surface antigen.

We observed HBsAg-positive samples in all age groups except the 80–89-year-olds. However, none of the samples in the age groups 0–9, 20–29, 30–39 and > 90 years were positive for both HBsAg and anti-HBc (Figure 2). Eleven of the 14 double-positive samples were observed in the age groups 40–79 years, with the highest number in the age group 60–69 years (Figure 2). Notably, three of the double-positive samples were observed in the age group 10–19 years, with respective ages of 16 (male), 17 (male), and 19 years (female). Males represented the majority of the HBsAg anti-HBc double-positive population (10/14). However, there was no statistically significant difference between sexes (p = 0.14).

The annotations indicate the number of respective samples. The age group 10–19 years was oversampled.

**Figure 3:**
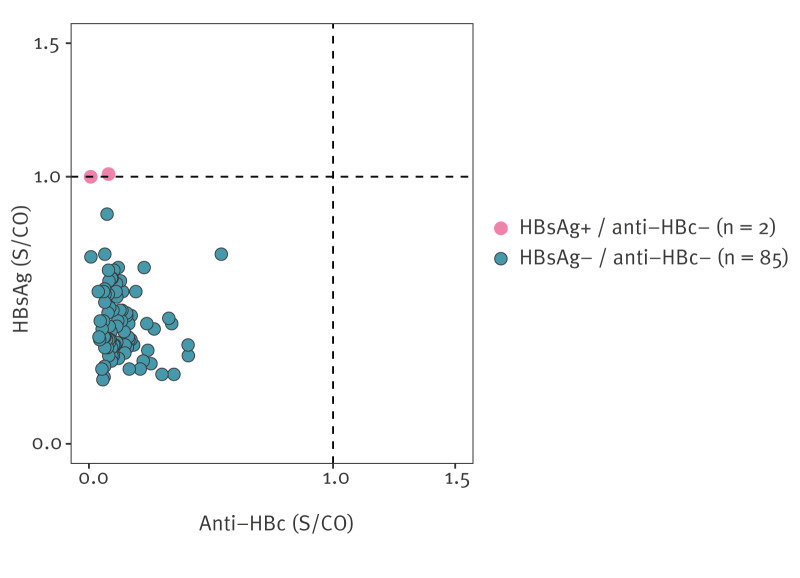
Signal-to-cutoff values of double-negative and HBsAg-positive anti-HBc-negative samples in ≤ 5-year-olds, Belgium, 2020 (n = 87)

Anti-HBc: hepatitis B core antibodies; HBsAg: hepatitis B surface antigen; S/CO: signal-to-cutoff.

A total of 87 (1.8%) samples originated from children aged 5 years or younger. Of these, two samples (2.3%), obtained from a 1-year-old and a 5-year-old, were HBsAg-positive, with respective S/CO values of 1.0 and 1.01 (Figure 3). Importantly, both samples were anti-HBc-negative in the immunoassay and had no specific anti-HBc signal in the neutralisation assay.

**Figure 4:**
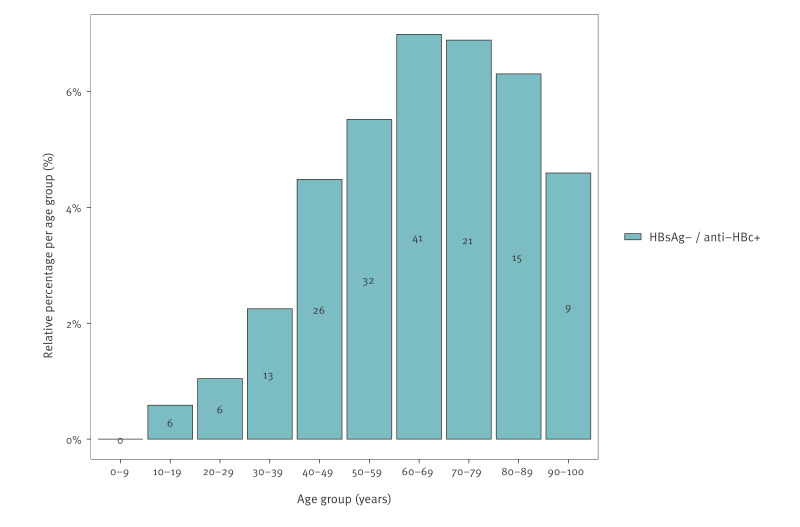
HBsAg-negative anti-HBc-positive samples across age groups in 2020, Belgium 2020 (n = 169)

Anti-HBc: hepatitis B core antibodies; HBsAg: hepatitis B surface antigen.

We observed HBsAg-negative anti-HBc-positive samples in all age groups except for the youngest age group (< 10 years-old) (Figure 4). The majority of these samples were from the older age groups, gradually increasing with increasing age. There was no statistically significant difference between sexes (p = 0.85).

The annotations indicate the number of respective samples. The age group 10–19 years was oversampled.

### Belgian hepatitis B virus prevalence estimates

Weighted analysis estimated an HBsAg seroprevalence of 0.74% (95% confidence interval (CI): 0.50–1.04) in Belgium in 2020. Importantly, when considering HBsAg and anti-HBc double positivity as criterion for HBV infection, an estimated national HBV seroprevalence of 0.25% (95% CI: 0.13–0.42) was retained (Table 3). In addition, we estimated that 3.59% (95% CI: 3.01–4.25) of the Belgian population in 2020 had previous exposure to HBV, as evidenced by an HBsAg-negative anti-HBc-positive serology (Table 3).

**Table 3:** Weighted hepatitis B virus prevalence estimates for the Belgian population, Belgium, 2020 (n = 4,954)

	Belgian population	
	%	95% CI
HBsAg+	0.74	0.50–1.04
HBsAg+ / anti-HBc+	0.25	0.13–0.42
HBsAg− / anti-HBc+	3.59	3.01–4.25

Anti-HBc: hepatitis B core antibodies; CI: confidence interval; HBsAg: hepatitis B surface antigen.

One sample with missing information about the postal code is omitted from the weighted analyses.

### Seroprevalence-based evaluation of the Belgian hepatitis B virus vaccination policy

The estimated HBsAg prevalence did not differ significantly between ≤ 33-year-olds – i.e. those born in or after 1987 and subject to the universal vaccination policy if born in Belgium – and the older age group (0.64%; 95% CI: 0.33–1.09 vs 0.80%; 95% CI: 0.48–1.24; p = 0.54). In contrast, when applying the double HBsAg and anti-HBc positivity criterion, the HBV prevalence was significantly lower in the vaccinated cohort (0.079%; 95% CI: 0.019–0.21) compared with the not universally vaccinated population aged > 33 years (0.36%; 95% CI: 0.17–0.64; p = 0.015). Similarly, the weighted estimate of past HBV exposure, indicated by an HBsAg-negative anti-HBc-positive serology, was significantly lower in the vaccinated cohort than in the not universally vaccinated cohort (0.68%; 95% CI: 0.37–1.13 vs 5.49%; 95% CI: 4.56–6.53; p < 0.001). 

## Discussion

This nationwide population-based serosurvey demonstrates the importance of combined HBsAg anti-HBc testing for the precise estimation of HBV seroprevalence by systematically analysing these markers in residual sera collected in three time periods in 2020. In addition, we here provided an updated weighted HBV seroprevalence for Belgium.

HBsAg was detected in 37 of the 4,955 samples (0.75%). Notably, almost two thirds (23/37) of the HBsAg-positive samples had an anti-HBc S/CO < 1.0, indicating non-reactivity. Early acute HBV infections can transiently manifest with a HBsAg-positive anti-HBc-negative serology, before genuine anti-HBc seroconversion. In rare cases, severely immunocompromised patients can fail to produce anti-HBc altogether, as has been described for haematological malignancies, other oncologic conditions and solid organ transplant recipients [22-24]. More frequently, transient low-level HBsAg seropositivity has been observed following recent HBV vaccination, particularly within 14 days [25], and up to 4 weeks in haemodialysis patients [26]. In addition, false-positive HBsAg results are often observed in automated commercial analysers, owing to their very high sensitivity, which can be caused e.g. by non-specific binding, cross-reactivity, the presence of heterophilic antibodies or human anti-animal antibodies, or sample contamination or degradation [16,27]. Nonetheless, these scenarios often yield low HBsAg S/CO values, typically > 1 and ≤ 20, as illustrated by Rysgaard et al. [25]. In our serosurvey, the majority (21/23) of the discordant HBsAg-positive anti-HBc-negative samples fell within this grey zone. The discordant samples which were above the grey zone had respective HBsAg S/CO values of 64.4 and 65.5, which remains remarkably lower than HBsAg levels of chronic HBV patients which typically exceed 1,000 S/CO. Although S/CO ratios provide useful insight in the differences between discordant and double-positive samples, an optimal cut-off has yet to be established. As thresholds are likely to be assay- or laboratory-specific and beyond the scope of this study, raw S/CO values should be interpreted with caution in combination with anti-HBc results.

It is highly unlikely that the discordant samples were obtained from haemodialysis or severely immunocompromised patients, as they originated from ambulatory primary care patients. In addition, vaccine-induced HBsAg positivity seems unlikely given that universal infant vaccination has been part of the Belgian vaccination policy since 1999 and we saw discordant samples in nearly all age categories, except for 80–89-year-olds. As there was insufficient sample volume left to perform a commercial confirmatory HBsAg assay or HBV DNA PCR, we opted to test the discordant samples in an anti-HBc neutralisation assay. Consistently, none of the HBsAg-positive anti-HBc-negative discordant samples showed a specific anti-HBc signal in the confirmatory neutralisation assay. Overall, this indicates that the HBsAg-positive results in the discordant anti-HBc-negative samples probably represent false positives.

In this serosurvey, we collected a total of 87 (1.8%) samples from children 5 years or younger. Among these, two samples (2.3%), originating from a 1- and a 5-year-old, tested positive for HBsAg, with S/CO values of 1.0 and 1.01 respectively. In addition, both samples were anti-HBc-negative and had no specific anti-HBc signal in the neutralisation assays. Given that both S/CO values are close to the cutoff, these discordant results probably represent false positives or vaccine carryover for the sample of the 1-year-old, as the final infant HBV vaccine dose is given at 15 months of age. As such, although based on a small number of samples, this subgroup analysis suggests that Belgium is on track to achieve the WHO impact target of HBV prevalence ≤ 0.1% in ≤ 5-year-olds.

Of note, three individuals aged 16, 17 and 19 years tested positive for both HBsAg and anti-HBc, despite their theoretical eligibility for infant vaccination in Belgium. A possible explanation is that these younger individuals were not born in Belgium. According to the Belgian Statistics Bureau (STATBEL), as of 1 January 2021, 2.04 million people (17.7% of the total population) living in Belgium were born outside the country, with a substantial number from high-endemic regions like Africa (25.9%) and Asia (16.6%) [28]. Our previous research demonstrated a high HBsAg prevalence of 6.8% and 3.4% (95% CI: 2.17–5.05), respectively in outreach screenings in Asian communities and civic integration programmes [29,30]. In addition, higher HBsAg prevalence has been reported in foreign-born blood donors [31]. These observations are consistent with findings from the Netherlands and Germany, where first-generation migrants respectively accounted for 81% and 58% of HBV infections [9,32]. Based on population registries and the respective postal codes of the three double HBsAg- and anti-HBc-positive individuals, their likelihood of being born outside of Belgium is 6.6%, 13.6% and 47.7%. Nonetheless, it is also possible that these individuals were vaccinated but failed to develop a protective immune response, leaving them susceptible to HBV infection. In this scenario, their infection would not necessarily be attributable to being born outside Belgium.

This serosurvey demonstrates how alternative criteria for HBV infection result in significantly different prevalence estimates. Using HBsAg positivity alone, the weighted HBV seroprevalence estimate for Belgium was 0.74% (95% CI: 0.50–1.04), whereas an HBV seroprevalence of 0.25% (95% CI: 0.13–0.42) was retained when we applied HBsAg anti-HBc double positivity as criterion. The implication of applying the correct criterion is particularly relevant for low-endemic countries like Belgium, as a few false-positive HBsAg results substantially inflate the prevalence estimates.

In addition, the results using HBsAg positivity alone would imply that the HBV prevalence in Belgium has remained unchanged since 2003 (HBsAg+: 0.66%; 95% CI: 0.51–0.84) [33] and 1997 (HBsAg+: 0.7%; 95% CI: 0.5–1.0) [34], despite the introduction of a universal vaccination policy in 1999 targeting all infants and performing a catch-up vaccination of the 12-year-olds. As such, all persons born in Belgium in or after 1987 were subject to this universal vaccination policy. Furthermore, HBsAg positivity would suggest that the weighted prevalence in the age cohort subject to the universal vaccination policy (≤ 33 years-old) would not significantly differ from the prevalence in the older age cohort, despite consistent high HBV vaccine coverage rates of ≥ 96.8% since 2012 and ≥ 90% since 2007 [35]. In contrast, the impact of the vaccination policy is evident when applying the double HBsAg anti-HBc positivity criterion, yielding significantly lower HBV prevalence estimates in the ≤ 33-year-olds compared with the > 33-year-olds (0.079% vs 0.36%, p = 0.015, respectively). The smaller number of past HBV infections in the younger age cohorts further supports the impact of Belgium’s vaccination policy.

In January 2025 the Belgian National Reference Centre for viral hepatitis (Sciensano) published a report on the epidemiological surveillance of HBV, based on reimbursed HBV-specific tests and voluntary reports from microbiology laboratories. In 2022, the number of individuals who received reimbursement for an HBV DNA test for the first time per person screened for HBsAg was 0.33% in Belgium, which could serve as a proxy for HBV prevalence, since testing is generally limited to HBsAg-positive individuals [36]. In addition, they describe that the majority (72%) of HBV diagnoses were in the age cohort which was not subject to the vaccination policy. While that report has several limitations (reliance on administrative data, inclusion of hospital laboratories that may overrepresent HBV patients, incomplete subsequent HBV DNA testing in HBsAg positives), the 0.33% proxy of HBV prevalence for 2022 broadly aligns with the prevalence estimate of our study.

Using the double positivity criterion, our weighted HBV prevalence estimate of 0.25% for the Belgian population in 2020 is lower than the Polaris Observatory 2022 modelled estimate of 0.5% (95% uncertainty interval (UI): 0.4–0.6) [37]. With our updated estimate, the HBV prevalence is comparable with those of neighbouring countries Germany (0.47%; 95% CI: 0.47–0.48%), France (0.30%: 95% CI: 0.13–0.70%), and the Netherlands (0.25%; 95% CI: 0.25–0.25%) (based on the most recent (2024) surveillance report of the European Centre for Disease Prevention and Control) [38-40]. The similarity can be explained by the fact that France and Germany implemented universal infant vaccination in 1995, around the same time as Belgium. The Netherlands introduced universal vaccination only in 2011, yet no clear prevalence differences are seen. Moreover, these countries have a similar migration pattern. The HBV prevalence in Belgium is notably lower than the 2019 estimates for the WHO European region (HBsAg+: 1.1%; 95% UI: 1.0–1.2) and other countries with a high socio-demographic index (HBsAg+: 1.5%; 95% UI: 1.4–1.6) [5]. In addition, these results imply that Belgium has reached a very low HBV endemicity (< 0.5%), comparable to northern European countries [14].

The findings of this study are strengthened by the large sample population, systematic testing strategy and prospective cross-sectional design. However, several limitations should be acknowledged. The lack of detailed socio-demographic data such as migration background, ethnicity and vaccination history precludes in-depth investigation of risk factors for HBV infection and the identification and quantification of vulnerable subpopulations. Retesting HBsAg-positive samples with an alternative commercial assay from a different manufacturer could be a useful confirmatory strategy, particularly in clinical practice where repeat testing and follow-up samples are feasible. However, as most commercial HBsAg assays rely on similar analytical principles and target comparable epitopes they could reproduce the same false-positive reactivity, especially in the presence of interfering substances. We therefore prioritised anti-HBc neutralisation as a confirmatory approach within the constraints of limited residual sample volume available for a HBsAg neutralisation assay or HBV DNA PCR. 

Although this study included weighting for national estimates, we cannot exclude potential underrepresentation of certain high-risk groups such as men who have sex with men, people who inject drugs, incarcerated people, sex workers or undocumented migrants. While their exact proportion in the population is uncertain, substantial deviations in HBV prevalence within these groups could influence the national estimate, highlighting the need for targeted sampling in future serosurveys to better capture these populations. In addition, the prevalence estimates were not formally adjusted for the diagnostic uncertainty of the HBsAg and anti-HBc assays. Moreover, although we specifically analysed samples from periods without SARS-CoV-2 lockdown restrictions, it remains possible that certain groups had an altered health-seeking behaviour.

## Conclusion

This nationwide population-based serosurvey highlights the critical importance of additional anti-HBc testing in HBsAg-positive samples to ensure correct and accurate estimation of HBV prevalences. Our results demonstrate how discordant serologies can substantially inflate and overestimate HBV prevalence, particularly in low-endemic settings, and obscure the impact of vaccination programmes. Therefore, we advocate to include HBsAg anti-HBc double positivity instead of HBsAg positivity alone as criterion for HBV infection in large population-based serosurveys, irrespective of the HBsAg S/CO level. This improves estimate reliability and correctness and helps distinguish likely false-positive HBsAg results from true infections, as anti-HBc is rarely negative in genuine HBV infection. This strategy relies on widely available, low-cost serological assays with limited sample volume requirements, thereby bypassing the need for expensive HBsAg neutralisation assays that require a large sample volume and are not widely available or impractical for large-scale serosurveys. Finally, we provide an updated national HBV prevalence estimate for Belgium to support country-level validation of hepatitis B control under the WHO European Region Action plan, and to inform and monitor other indicators for HBV elimination.

## Data Availability

The data that support the findings of this study are available from the corresponding author, TV, upon reasonable request.

## Supplementary Data

140000 Supplement
