# Supplementary material for: Combined HBsAg and anti-HBc testing is required to estimate hepatitis B virus seroprevalence in a low-endemic country: findings from a nationwide population-based serosurvey, Belgium, 2020
Source: Euro Surveill. 2026 Feb 12;31(6):2500533. doi: 10.2807/1560-7917.ES.2026.31.6.2500533 (PMC12905530; doi:10.2807/1560-7917.ES.2026.31.6.2500533)
Supplement: Supplement [file 25-00533_VANWOLLEGHEM_Supplement.pdf]

Combined HBsAg and anti-HBc testing is required to estimate HBV seroprevalence in a low-endemic country: findings from a nationwide population-based serosurvey in Belgium

THIS SUPPLEMENTARY MATERIAL IS HOSTED BY EUROSURVEILLANCE AS SUPPORTING INFORMATION ALONGSIDE THE ARTICLE [COMBINED HBSAG AND ANTI-HBC TESTING IS REQUIRED TO ESTIMATE HBV SEROPREVALENCE IN A LOW-ENDEMIC COUNTRY: FINDINGS FROM A NATIONWIDE POPULATION-BASED SEROSURVEY], ON BEHALF OF THE AUTHORS, WHO REMAIN RESPONSIBLE FOR THE ACCURACY AND APPROPRIATENESS OF THE CONTENT. THE SAME STANDARDS FOR ETHICS, COPYRIGHT, ATTRIBUTIONS AND PERMISSIONS AS FOR THE ARTICLE APPLY. SUPPLEMENTS ARE NOT EDITED BY EUROSURVEILLANCE AND THE JOURNAL IS NOT RESPONSIBLE FOR THE MAINTENANCE OF ANY LINKS OR EMAIL ADDRESSES PROVIDED THEREIN.

TABLE OF CONTENTS

FIGURE S1 3

Figure S1: Comparison of the sample population and the Belgian population in 2020.
